# Supplementary material for: Direction of attentional focus in prosthetic training: Current practice and potential for improving motor learning in individuals with lower limb loss
Source: PLoS One. 2022 Jul 7;17(7):e0262977. doi: 10.1371/journal.pone.0262977 (PMC9262185; doi:10.1371/journal.pone.0262977)

**Appendix**

Appendix A: Example of a Completed Analysis Matrix: Focus of Attention

The analysis matrix below was used to analyze the recorded video from each participant. Each block represented 1-minute blocks in the recorded video. For instance, “Minute 1” was between zero to 60 seconds of the recorded video. Within each time block, a single tally was represented by “I” and showed a single incidence of a particular instruction or feedback made by the prosthetist to the participant. The shaded blocks indicated which tasks were completed during a time block.


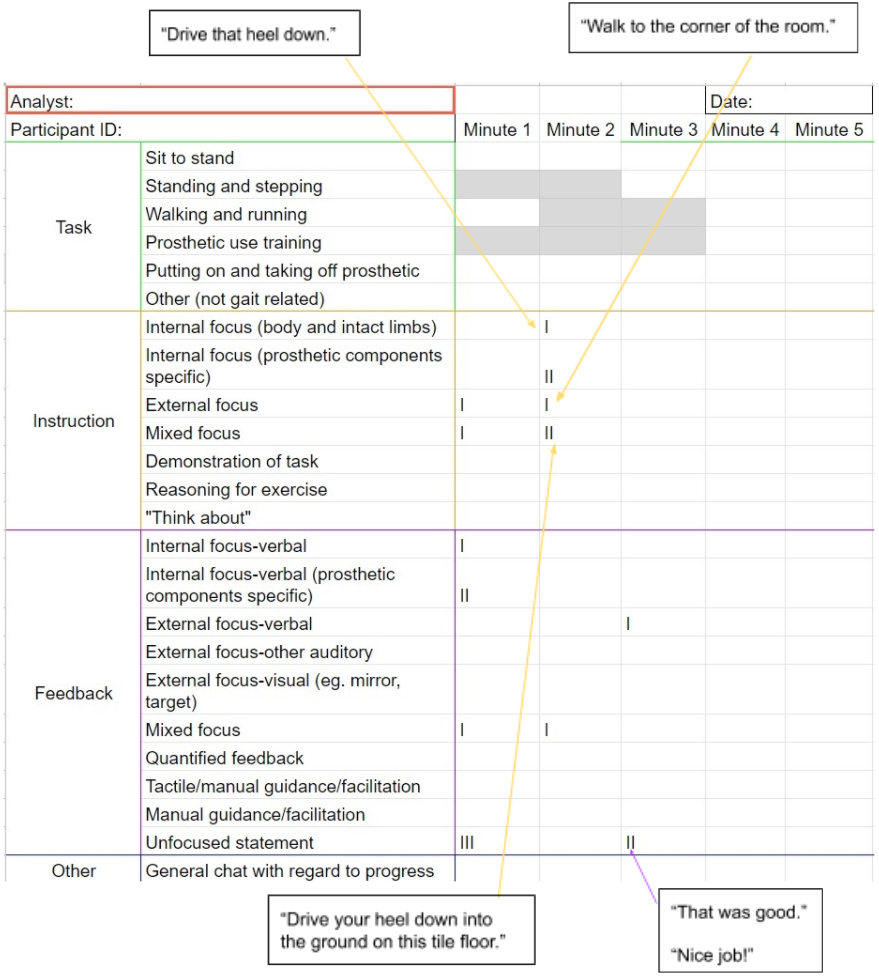

Supplement: S1 Appendix — (DOCX) [file pone.0262977.s002.docx]
