# Supplementary material for: Direction of attentional focus in prosthetic training: Current practice and potential for improving motor learning in individuals with lower limb loss
Source: PLoS One. 2022 Jul 7;17(7):e0262977. doi: 10.1371/journal.pone.0262977 (PMC9262185; doi:10.1371/journal.pone.0262977)
Supplement: Attachment — Submitted filename: General comments.docx [file pone.0262977.s003.docx]

**General comments**

This study makes a novel attempt to describe the nature of instructional content delivered by prosthetists during rehabilitation sessions with lower limb prosthetic users. Interestingly, the results indicate a large bias towards verbal instructions that direct the patient’s attention towards the explicit monitoring and control of the body/prosthesis (i.e., internal focus). Such a finding corroborates with other fields of rehabilitation and acts as an extremely important point of reference for research moving forward. Indeed, there is substantial evidence that such instructions can be detrimental to motor control and hinder rehabilitation process. It is hoped that these findings drive future research to tackle this potential flaw in current practices.

In general, I applaud the authors for an extremely well-written paper that adequately highlights the importance of the work and its possible applications. The methodology is extremely thorough and well thought-out, overcoming many limitations that are typically associated with this type of research. I don’t have any huge concerns with the paper, but I do have many points that I would like the authors to address and clarify. In particular, I feel the statistical process could be clearer as I found myself somewhat confused with the components of your regression model and ANOVA. I also feel the authors could reshape their discussion to ensure their key findings are presented prior to the discussion of additional findings that were perhaps an afterthought throughout. The discussion is also somewhat lengthy and repetitive in places, but this is perhaps a preference of my own rather than a necessary change. I recommend several studies that could be used to strengthen the breadth and rationale of this study.

I thoroughly enjoyed reading this work. Thanks!

**Minor comments**

***Introduction***

L11:17 – Your opening section does a great job of highlighting the need for evidence-based practice. A recent review paper by Parr et al. (2021) also highlights the importance of applying motor learning principles to prosthesis rehabilitation, including a specific section on attentional focus. Perhaps this review could be added to strengthen this section and highlight recent calls for research such as yours.

- *Parr, J. V., Wright, D. J., Uiga, L., Marshall, B., Mohamed, M. O., & Wood, G. (2021). A scoping review of the application of motor learning principles to optimize myoelectric prosthetic hand control. Prosthetics and Orthotics International.*

L18:32 - Perhaps this section is a little backwards - it might be clearer to first explain to the naive reader what external vs internal instructions are, before exploring the findings of relevant literature. Knowledge of these terms is a little assumed.

L42:45 - This is an important statement, but I think you need to briefly explain *WHY* it is agreed. What exactly are the mechanisms underpinning the benefits of an external focus (e.g., Poolton & Maxwell, 2006)? For prosthetics, two recent studies have explored and discussed the benefit of achieving an external focus via “serious gaming” (Kristoffersen et al., 2020, 2021), thus reinforcing the need for application to lower limb control. From a mechanistic point of view, a study in upper-limb prosthetics by Parr et al. (2019) found benefits of adopting an external focus via gaze training and proposed such benefits might be supported by lower cognitive demands, unfreezing the degrees of freedom, and lowering the tendency for engaging in conscious motor processing. These points could strengthen your rationale.

- *Poolton, J. M., Maxwell, J. P., Masters, R. S. W., & Raab, M. (2006). Benefits of an external focus of attention: Common coding or conscious processing?. Journal of sports sciences, 24(1), 89-99.*
- *Parr, J. V. V., Vine, S. J., Wilson, M. R., Harrison, N. R., & Wood, G. (2019). Visual attention, EEG alpha power and T7-Fz connectivity are implicated in prosthetic hand control and can be optimized through gaze training. Journal of neuroengineering and rehabilitation, 16(1), 1-20.*
- *Kristoffersen, M. B., Franzke, A. W., Van Der Sluis, C. K., Murgia, A., & Bongers, R. M. (2020). Serious gaming to generate separated and consistent EMG patterns in pattern-recognition prosthesis control. Biomedical Signal Processing and Control, 62, 102140.*
- *Kristoffersen, M. B., Franzke, A. W., Bongers, R. M., Wand, M., Murgia, A., & van der Sluis, C. K. (2021). User training for machine learning controlled upper limb prostheses: a serious game approach. Journal of NeuroEngineering and Rehabilitation, 18(1), 1-15.*

L52:53 – Has your hypothesis regarding the frequency of internal focus instructions been justified enough here. Indeed, I believe there is plenty of research available to lead you towards this conclusion that could be used to pre-empt this statement. Two recent studies by Kristoffersen et al. (2020, 2021) have also made this point and might be worth checking.

- *Kristoffersen, M. B., Franzke, A. W., Van Der Sluis, C. K., Murgia, A., & Bongers, R. M. (2020). Serious gaming to generate separated and consistent EMG patterns in pattern-recognition prosthesis control. Biomedical Signal Processing and Control, 62, 102140.*
- *Kristoffersen, M. B., Franzke, A. W., Bongers, R. M., Wand, M., Murgia, A., & van der Sluis, C. K. (2021). User training for machine learning controlled upper limb prostheses: a serious game approach. Journal of NeuroEngineering and Rehabilitation, 18(1), 1-15.*

L56:57 – Again, I am not sure this hypothesis has been fully justified. Could the authors elaborate on how they fell upon this? Is there any research you could briefly introduce?

***Methods***

L74:75 – An interesting exclusion. I wonder how pre-prosthetic instructions affect subsequent prosthesis interaction.

L113:114 – Perhaps the reader would benefit from the other two types of instruction being defined here?

L131:133 - A bit more detail is needed here...

I believe the Kruskal-Wallis is an independent ANOVA? But from what I understand your IV here is "instruction" for which each prosthetist would have 3 values. Would this not require a repeated measures ANOVA of sorts? Am I right in thinking that here you are comparing the 6 prosthetists? If so, then were scores aggregated for a single prosthetist when they have multiple pairings? Or have you actually included 21 prosthetist-patient pairs in this analysis? If so, this is a little tricky because technically your sample isn't independent as the same prosthetist would have contributed to several observations? Some clarification on this process would be appreciated.

L135:140 – Unfortunately I am not too familiar with the Tobit model. However, I found it difficult to understand exactly how your model was structured. From what I understand, you performed three separate regression models with each instruction type (internal, external, mixed) acting as the dependent variable for each model? Prosthetist’s experience and LLP characteristic variables were then used as a selection of predictor variables? If so, I think you can make this clearer to the reader. Also, I believe this process would involve a high number of separate bivariate regressions to be performed. Did the authors do anything to control for the inflated error rate?

L175:177 – Again to clarify, does this mean there was no difference in the frequency of instruction type across the six prosthetists? In other words, your IV is instruction type (internal, external, mixed) for which you have 6 observations each? This seems a little contradictory to the finding of a high bias towards internal instructions reported later. I apologise if I am misunderstanding.

***Discussion***

General - I might suggest a re-structuring of your discussion. Your key finding (bias towards internal) could be discussed first to highlight its importance. The findings regarding the biases should come later as they are less central to your paper.

L226:227 - These findings are very interesting. However, I'm wondering if the authors provide enough (if any) justification for running this analysis in the first place. It would also be meaningful to elaborate on these findings a little and provide some evidence-based speculation (if possible) to better set-up future investigations into these biases. At the moment, these analyses feel rather throw-away.

L229:231 - Could the authors delve into the rehab/sporting literature to find evidence that might support these claims? I believe there is ample evidence that coaches do indeed tend to refine subtle movement corrections in the search for the “perfect technique”. Maybe this could be used to improve the breadth of these findings?

L252:254 - Not sure these need to be defined again. A clearer definition in introduction as suggested would perhaps suffice by this point.

L286:289 – This is an interesting point and sounds a lot like an analogy! Indeed, such instructions have been applied to the re-learning of gait in Parkinson’s. For example, Jie et al. () told PD patients to pretend they were “following footprints in the sand” which led to rehabilitation gains. The review paper by Parr et al. (2021) covers the application of analogy learning for prosthesis rehabilitation and might be worth checking.

- *Jie, L. J., Goodwin, V., Kleynen, M., Braun, S., Nunns, M., & Wilson, M. (2016). Analogy learning in Parkinson's disease: a proof-of-concept study. International Journal of Therapy and Rehabilitation, 23(3), 123-130.*
