# Supplementary material for: Direction of attentional focus in prosthetic training: Current practice and potential for improving motor learning in individuals with lower limb loss
Source: PLoS One. 2022 Jul 7;17(7):e0262977. doi: 10.1371/journal.pone.0262977 (PMC9262185; doi:10.1371/journal.pone.0262977)
Supplement: Attachment — Submitted filename: Response to authors.docx [file pone.0262977.s005.docx]

Response to authors

I thank the authors for making a great attempt to address my concerns. The structure and flow of the paper is much improved. However, there is still a more detail I would like in places, particularly regarding the analyses. I feel some of the responses have somewhat passively addressed my concerns and I worry that there is a misinterpretation of non-significant results. I would therefore appreciate further elaboration upon the following points:

Authors:

*“As the focus of this study was observational and on lower limb prosthesis, we decided to put the Kristoffersen et al. studies in the discussion section to avoid overwhelming the readers, particularly clinicians, in the introduction. The following two references were added to line 270-273 in the discussion to highlight the need to understand the science underlying prosthetic training in response to the more sophisticated control requirements of modern prostheses.”*

RESPONSE:

My focus here was not primarily to highlight the Kristoffersen paper here per se. Rather, I was hoping the authors could provide some evidence in general to support the prediction that internal focus instructions would be highly frequent during rehabilitation. The authors make a good attempt to explain why internal focus instructions might be less effective, but little to suggest this practice would be common. I again feel as though the rationale for the study and the justification for your hypotheses could be strengthened by literature on the instructional content of rehabilitation practice. For example, is there any subjective data from therapists or patients across any domains that suggests a bias towards an internal focus? Indeed, you actually provide this information in the discussion. Could it be brought forward?

Authors:

*“Our primary justification for the study was that instructions to prosthetic device users may impact how well they learn to use the device. Clinical application of this theory has been demonstrated in several rehabilitation studies, which were cited here. To more clearly delineate our intention, we revised this sentence (line 46-49) to the following:”*

RESPONSE:

I believe my initial response was slightly too vague and therefore remains somewhat unanswered. To be specific, I want to know why the authors think IF instructions would be utilised less with higher function LLP users? Is there a specific rationale for this hypothesis? Currently it doesn’t seem justified. Are you suggesting that high functioning LLP users are more skilled ***because*** they are not receiving If instructions? Or are you suggesting that IF instructions are used more at the early stage of learning and less so when an LLP users becomes more skills? Either of these explanations need justification. Again, these points are better addressed in the discussion and therefore reads as though the hypotheses were derived post-hoc.

Authors:

*We provided the definitions and examples for “mixed focus” and “unfocused” statements in Table 1. Specifically, mixed focus was defined as: “A statement that includes both internal and external focus”, and unfocused statement was defined as: “A statement not giving technical instruction or offering encouragement to the learner only.”*

RESPONSE:

I was aware of the definitions in Table 1 but found it odd that only one type of instruction was defined in text. Is there a reason for this? Upon first reading I felt as though I would have benefited from all being defined in the same section.

Authors:

*Thank you for the suggestion. The Kruskal-Wallis tests were done to compare among the 6*

*prosthetists to see if there were any significant differences in their uses of internal and external*

*languages. The percentage for each type of statement used was aggregated for each prosthetist.*

RESPONSE:

Unfortunately, I am more confused now! If each type of statement were aggregated for each prosthetist, then you must have three values for each of the six prosthetists, one for IF, EF and mixed? You are then running the ANOVA to determine if there is a main effect of instruction type, and whether the group of six prosthetist tend to use one type of instruction more than another. If this is the case, then your design is technically repeated measures, and the Kruskal-Wallis is not suitable?

Alternatively, I don’t understand how you can run an analyses that compares each prosthetists individually to determine whether one prosthetist might use a type of instruction more than another?

I would need much more clarity here.

Author:

*Thank you for the suggestion. The Kruskal-Wallis tests were done to compare among the 6*

*prosthetists to see if there were any significant differences in their uses of internal and external*

*languages. The percentage for each type of statement used was aggregated for each prosthetist.*

*We revised this statement to the following to clarify:*

*“The nonparametric Kruskal-Wallis test was used to determine whether there was a significant difference in the attentional focus statement types delivered by the six participating prosthetists in the study.”*

*The revised sentence above now exactly corresponds with the following sentence in the results*

*section:*

*“There were no statistical differences in the types of attentional focus statements delivered by*

*the six prosthetists during the training sessions (p=0.330-0.945).”*

RESPONSE:

So to confirm, there was no significant different in the type of instruction delivered? This would suggest that IF instructions were not used any more frequently than EF and mixed instructions? Although the descriptive statistics suggest a bias towards IF instructions you inferential statistics do not? Unless I am mistaken this would prevent you from drawing the conclusion that

“Our hypothesis was confirmed that most of the verbal interactions delivered by prosthetists to LLP users were focused internally on the movements of the patients’ body and/or prosthesis, rather than externally on the intended movement effects.”

and

“Our results showed **a significant** but perhaps unsurprising trend that most (i.e. 63%)…”

Please can the authors explain this discrepancy?

*Authors:*

*This secondary analysis was done to examine whether certain characteristics of the participants*

*(clinicians and patients) were predictive of greater use of the presumably less effective internal*

*focus instruction and feedback. We move this discussion to after the primary finding discussion,*

*and added the following sentence:*

RESPONSE:

I still feel the authors could attempt to speculate their finding regarding their finding that age and gender were related to frequency of IF. This would help better set up future work.
