# Supplementary material for: Direction of attentional focus in prosthetic training: Current practice and potential for improving motor learning in individuals with lower limb loss
Source: PLoS One. 2022 Jul 7;17(7):e0262977. doi: 10.1371/journal.pone.0262977 (PMC9262185; doi:10.1371/journal.pone.0262977)
Supplement: Attachment — Submitted filename: Response to reviewer comments v1.docx [file pone.0262977.s004.docx]

**General comments**

This study makes a novel attempt to describe the nature of instructional content delivered by prosthetists during rehabilitation sessions with lower limb prosthetic users. Interestingly, the results indicate a large bias towards verbal instructions that direct the patient’s attention towards the explicit monitoring and control of the body/prosthesis (i.e., internal focus). Such a finding corroborates with other fields of rehabilitation and acts as an extremely important point of reference for research moving forward. Indeed, there is substantial evidence that such instructions can be detrimental to motor control and hinder rehabilitation process. It is hoped that these findings drive future research to tackle this potential flaw in current practices.

In general, I applaud the authors for an extremely well-written paper that adequately highlights the importance of the work and its possible applications. The methodology is extremely thorough and well thought-out, overcoming many limitations that are typically associated with this type of research. I don’t have any huge concerns with the paper, but I do have many points that I would like the authors to address and clarify. In particular, I feel the statistical process could be clearer as I found myself somewhat confused with the components of your regression model and ANOVA. I also feel the authors could reshape their discussion to ensure their key findings are presented prior to the discussion of additional findings that were perhaps an afterthought throughout. The discussion is also somewhat lengthy and repetitive in places, but this is perhaps a preference of my own rather than a necessary change. I recommend several studies that could be used to strengthen the breadth and rationale of this study.

I thoroughly enjoyed reading this work. Thanks!

**Thank you for the kind words. We appreciate your constructive comments, and have carefully prepared a response below. All changes in the revised manuscript is highlighted in yellow. Your review made the manuscript richer and clearer!**

**Minor comments**

***Introduction***

L11:17 – Your opening section does a great job of highlighting the need for evidence-based practice. A recent review paper by Parr et al. (2021) also highlights the importance of applying motor learning principles to prosthesis rehabilitation, including a specific section on attentional focus. Perhaps this review could be added to strengthen this section and highlight recent calls for research such as yours.

- *Parr, J. V., Wright, D. J., Uiga, L., Marshall, B., Mohamed, M. O., & Wood, G. (2021). A scoping review of the application of motor learning principles to optimize myoelectric prosthetic hand control. Prosthetics and Orthotics International.*

**Thank you for providing the reference of this recently published scoping review. We added it here to highlight the need to incorporate motor learning principles in prosthetic training.**

L18:32 - Perhaps this section is a little backwards - it might be clearer to first explain to the naive reader what external vs internal instructions are, before exploring the findings of relevant literature. Knowledge of these terms is a little assumed.

**We appreciate the suggestion and revised this paragraph to explain the internal vs. external focus before giving the research example. The following was added:**

**“**This theory states that by instructing learners to focus on controlling the motions of body segments or muscle contractions (i.e. internal focus), it may interfere with the natural motor control processes. In contrast, an external focus of attention on the intended movement outcomes allows the learner to adopt a more automatic form of control and is therefore more effective.(1-3)”

L42:45 - This is an important statement, but I think you need to briefly explain *WHY* it is agreed. What exactly are the mechanisms underpinning the benefits of an external focus (e.g., Poolton & Maxwell, 2006)? For prosthetics, two recent studies have explored and discussed the benefit of achieving an external focus via “serious gaming” (Kristoffersen et al., 2020, 2021), thus reinforcing the need for application to lower limb control. From a mechanistic point of view, a study in upper-limb prosthetics by Parr et al. (2019) found benefits of adopting an external focus via gaze training and proposed such benefits might be supported by lower cognitive demands, unfreezing the degrees of freedom, and lowering the tendency for engaging in conscious motor processing. These points could strengthen your rationale.

- *Poolton, J. M., Maxwell, J. P., Masters, R. S. W., & Raab, M. (2006). Benefits of an external focus of attention: Common coding or conscious processing?. Journal of sports sciences, 24(1), 89-99.*
- *Parr, J. V. V., Vine, S. J., Wilson, M. R., Harrison, N. R., & Wood, G. (2019). Visual attention, EEG alpha power and T7-Fz connectivity are implicated in prosthetic hand control and can be optimized through gaze training. Journal of neuroengineering and rehabilitation, 16(1), 1-20.*
- *Kristoffersen, M. B., Franzke, A. W., Van Der Sluis, C. K., Murgia, A., & Bongers, R. M. (2020). Serious gaming to generate separated and consistent EMG patterns in pattern-recognition prosthesis control. Biomedical Signal Processing and Control, 62, 102140.*
- *Kristoffersen, M. B., Franzke, A. W., Bongers, R. M., Wand, M., Murgia, A., & van der Sluis, C. K. (2021). User training for machine learning controlled upper limb prostheses: a serious game approach. Journal of NeuroEngineering and Rehabilitation, 18(1), 1-15.*

**We greatly appreciate the suggestion and the references. We added the Poolton et al. study to line 23 to explain why external focus is generally considered more natural and effective (see above). We also added the citations for Parr et al. 2019 and 2021 studies to highlight that improved learning strategy may also benefit prosthetic device acceptance (by reducing learning fatigue and frustration) in line 45-46.**

L52:53 – Has your hypothesis regarding the frequency of internal focus instructions been justified enough here. Indeed, I believe there is plenty of research available to lead you towards this conclusion that could be used to pre-empt this statement. Two recent studies by Kristoffersen et al. (2020, 2021) have also made this point and might be worth checking.

- *Kristoffersen, M. B., Franzke, A. W., Van Der Sluis, C. K., Murgia, A., & Bongers, R. M. (2020). Serious gaming to generate separated and consistent EMG patterns in pattern-recognition prosthesis control. Biomedical Signal Processing and Control, 62, 102140.*
- *Kristoffersen, M. B., Franzke, A. W., Bongers, R. M., Wand, M., Murgia, A., & van der Sluis, C. K. (2021). User training for machine learning controlled upper limb prostheses: a serious game approach. Journal of NeuroEngineering and Rehabilitation, 18(1), 1-15.*

**As the focus of this study was observational and on lower limb prosthesis, we decided to put the Kristoffersen et al. studies in the discussion section to avoid overwhelming the readers, particularly clinicians, in the introduction. The following two references were added to line 270-273 in the discussion to highlight the need to understand the science underlying prosthetic training in response to the more sophisticated control requirements of modern prostheses.**

“Prosthetic skill learning may be of even greater importance today owing to the proliferation of sophisticated prosthetic components that are designed and optimized to promote specific and complex movement patterns.(4, 5)”

- **Franzke AW, Kristoffersen MB, Bongers RM, Murgia A, Pobatschnig B, Unglaube F, et al. Users' and therapists' perceptions of myoelectric multi-function upper limb prostheses with conventional and pattern recognition control. PloS one. 2019;14(8):e0220899.**
- **Kristoffersen MB, Franzke AW, Bongers RM, Wand M, Murgia A, van der Sluis CK. User training for machine learning controlled upper limb prostheses: a serious game approach. Journal of neuroengineering and rehabilitation. 2021;18(1):32.**

L56:57 – Again, I am not sure this hypothesis has been fully justified. Could the authors elaborate on how they fell upon this? Is there any research you could briefly introduce?

**Our primary justification for the study was that instructions to prosthetic device users may impact how well they learn to use the device. Clinical application of this theory has been demonstrated in several rehabilitation studies, which were cited here. To more clearly delineate our intention, we revised this sentence (line 46-49) to the following:**

“Because of this emphasis on skill learning, practice, and motor adaptation, it is generally agreed that motor learning strategies including the adoption of an external focus of attention when delivering instructions or feedback to LLP users could benefit prosthetic training, device acceptance, and improve rehabilitation outcomes after amputation.(6, 7) It may be beneficial for prosthetists and other post-amputation care providers to recognize that the words used in their instructions pertaining to how to operate a prosthetic device can have a tangible impact on the patient’s learning, as has been demonstrated in other disciplines of rehabilitation.(8-13)”

***Methods***

L74:75 – An interesting exclusion. I wonder how pre-prosthetic instructions affect subsequent prosthesis interaction.

**We imposed this particular exclusion criterion because the purpose of the study was to observe the interactions between prosthetists and patients during prosthetic training involving movement instructions (i.e. gait training). Furthermore, in our analysis we quantified the amount of instruction and feedback statements pertaining to the patient’s body vs. prosthesis, so including some participants without a prosthesis (pre-prosthetic) would not have allowed this analysis.**

**I do agree that it would be interesting to see how instructions evolve over the course of post-amputation rehabilitation. Our data seem to indicate that higher functioning patients (higher PLUS-M score) tend to receive higher percentages of internal focus instructions and feedback. A future study direction!**

L113:114 – Perhaps the reader would benefit from the other two types of instruction being defined here?

**We provided the definitions and examples for “mixed focus” and “unfocused” statements in Table 1. Specifically, mixed focus was defined as: “A statement that includes both internal and external focus”, and unfocused statement was defined as: “A statement not giving technical instruction or offering encouragement to the learner only.”**

L131:133 - A bit more detail is needed here...

I believe the Kruskal-Wallis is an independent ANOVA? But from what I understand your IV here is "instruction" for which each prosthetist would have 3 values. Would this not require a repeated measures ANOVA of sorts? Am I right in thinking that here you are comparing the 6 prosthetists? If so, then were scores aggregated for a single prosthetist when they have multiple pairings? Or have you actually included 21 prosthetist-patient pairs in this analysis? If so, this is a little tricky because technically your sample isn't independent as the same prosthetist would have contributed to several observations? Some clarification on this process would be appreciated.

**Thank you for the suggestion. The Kruskal-Wallis tests were done to compare among the 6 prosthetists to see if there were any significant differences in their uses of internal and external languages. The percentage for each type of statement used was aggregated for each prosthetist.**

**We revised this statement to the following to clarify:**

**“**The nonparametric Kruskal-Wallis test was used to determine whether there was a significant difference in the attentional focus statement types delivered by the six participating prosthetists in the study.”

**The revised sentence above now exactly corresponds with the following sentence in the results section:**

**“**There were no statistical differences in the types of attentional focus statements delivered by the six prosthetists during the training sessions (p=0.330-0.945).”

L135:140 – Unfortunately I am not too familiar with the Tobit model. However, I found it difficult to understand exactly how your model was structured. From what I understand, you performed three separate regression models with each instruction type (internal, external, mixed) acting as the dependent variable for each model? Prosthetist’s experience and LLP characteristic variables were then used as a selection of predictor variables? If so, I think you can make this clearer to the reader. Also, I believe this process would involve a high number of separate bivariate regressions to be performed. Did the authors do anything to control for the inflated error rate?

**You are correct. The multi-variate, Tobit regression analysis was done to examine whether participating prosthetists’ years of experience, and/or patients’ characteristics (sex, age, years since amputation, cause of amputation, amputation level, and PLUS-M T-score) were associated with greater use of the presumably less effective internal focus instructions and feedback. Only one model analysis for internal focus was conducted because this focus type accounted for 63% of all statements. We clarified this analysis to the following:**

**“**Multi-variate regression analysis using the Tobit model was conducted to determine whether the prosthetists’ experience and/or LLP users’ characteristics (sex, age, years since amputation, cause of amputation, amputation level, and mobility measured by PLUS-M) were associated with greater use of internal focus instructions and feedback during prosthetic training.”

L175:177 – Again to clarify, does this mean there was no difference in the frequency of instruction type across the six prosthetists? In other words, your IV is instruction type (internal, external, mixed) for which you have 6 observations each? This seems a little contradictory to the finding of a high bias towards internal instructions reported later. I apologise if I am misunderstanding.

**We appreciate you pointing out potential areas of confusion so we can clarify them. As mentioned above (L131:133), we revised the descriptions of this comparison so the sentence in Data Analysis corresponds with the Results sentence:**

**Data analysis: “**The nonparametric Kruskal-Wallis test was used to determine whether there was a significant difference in the proportion of attentional focus statement types delivered by the six participating prosthetists.”

**Results: “**There were no statistical differences in the types of attentional focus statements delivered by the six prosthetists during the training sessions (p=0.330-0.945).”

***Discussion***

General - I might suggest a re-structuring of your discussion. Your key finding (bias towards internal) could be discussed first to highlight its importance. The findings regarding the biases should come later as they are less central to your paper.

**Thank you for the great suggestion. We agree that we should focus on discussing our primary finding (clinicians’ bias towards the greater use of internal focus statements), before going into the secondary findings (predictors of increased use of internal focus statements). As suggested, we re-structured the discussion so the sequence of the paragraphs flow more logically.**

L226:227 - These findings are very interesting. However, I'm wondering if the authors provide enough (if any) justification for running this analysis in the first place. It would also be meaningful to elaborate on these findings a little and provide some evidence-based speculation (if possible) to better set-up future investigations into these biases. At the moment, these analyses feel rather throw-away.

**This secondary analysis was done to examine whether certain characteristics of the participants (clinicians and patients) were predictive of greater use of the presumably less effective internal focus instruction and feedback. We move this discussion to after the primary finding discussion, and added the following sentence:**

**“**In speculation that LLP users with certain characteristics may be biased to receive a greater proportion of the presumably less effective internally focused instruction and feedback, our secondary analysis examined these associations.”

L229:231 - Could the authors delve into the rehab/sporting literature to find evidence that might support these claims? I believe there is ample evidence that coaches do indeed tend to refine subtle movement corrections in the search for the “perfect technique”. Maybe this could be used to improve the breadth of these findings?

**Thank you for raising this very interesting point. To answer this question, we looked up a number of studies on the use of attentional focus instructions in sports. We found that the use of internally-focused language is very common (up to 80+%) in top level athletes including elite youth baseball pitchers(14), NCAA division one athletes including soccer, golf, tennis, and volleyball(15), and elite track and field athletes(16). Comparatively, this percentage seems to be lower (50-60%) in reactional distance runners(17). However, it should be noted that these percentages are highly dependent on the sport disciplines, and currently there is no direct evidence on how athletes’ skill levels correlate with the types of attentional focus instructions they receive. In fact, our findings that amputee patient’s mobility level (as measured by PLUS-M) was positively correlated with the amount of received internally-focused instruction is perhaps the only available data on this question at this moment.**

**In a personal communication with Dr. Gabriele Wulf, a leading researcher on this topic, she is of the opinion that the idea of refining movement forms requires internal focus instructions is a misconception. It may be more “convenient” to use internal focus instructions to correct subtle movements, but that does not mean it is superior to individuals at any performance level.(18)**

**We included one additional reference to the revised statement (line 264-268):**

“It is possible that when treating higher functioning LLP users, prosthetists may choose to focus on addressing subtle body movements such as movement symmetry and sequence, which may lead to the more prevailing use of internally-focused instructions and feedback.(19) Nonetheless, these potential biases may impact the outcomes of patients at different stages of their recovery, and should be examined further in the future.”

- **Yamada M, Higgins LQ, Raisbeck L. How external and internal focus are used in the field: A review. Int J Sports Sci Coa. 2021.**

L252:254 - Not sure these need to be defined again. A clearer definition in introduction as suggested would perhaps suffice by this point.

**We deleted the redundant definitions here.**

L286:289 – This is an interesting point and sounds a lot like an analogy! Indeed, such instructions have been applied to the re-learning of gait in Parkinson’s. For example, Jie et al. () told PD patients to pretend they were “following footprints in the sand” which led to rehabilitation gains. The review paper by Parr et al. (2021) covers the application of analogy learning for prosthesis rehabilitation and might be worth checking.

- *Jie, L. J., Goodwin, V., Kleynen, M., Braun, S., Nunns, M., & Wilson, M. (2016). Analogy learning in Parkinson's disease: a proof-of-concept study. International Journal of Therapy and Rehabilitation, 23(3), 123-130.*

**Thank you, we added the Jie et al. study to corroborate our suggestion of using floor markers as external targets for gait training.**

**References:**

1.	Richer N, Saunders D, Polskaia N, Lajoie Y. The effects of attentional focus and cognitive tasks on postural sway may be the result of automaticity. Gait Posture. 2017;54:45-9.

2.	Wulf G, McNevin N, Shea CH. The automaticity of complex motor skill learning as a function of attentional focus. The Quarterly journal of experimental psychology A, Human experimental psychology. 2001;54(4):1143-54.

3.	Poolton JM, Maxwell JP, Masters RS, Raab M. Benefits of an external focus of attention: common coding or conscious processing? Journal of sports sciences. 2006;24(1):89-99.

4.	Franzke AW, Kristoffersen MB, Bongers RM, Murgia A, Pobatschnig B, Unglaube F, et al. Users' and therapists' perceptions of myoelectric multi-function upper limb prostheses with conventional and pattern recognition control. PloS one. 2019;14(8):e0220899.

5.	Kristoffersen MB, Franzke AW, Bongers RM, Wand M, Murgia A, van der Sluis CK. User training for machine learning controlled upper limb prostheses: a serious game approach. Journal of neuroengineering and rehabilitation. 2021;18(1):32.

6.	Parr JVV, Vine SJ, Wilson MR, Harrison NR, Wood G. Visual attention, EEG alpha power and T7-Fz connectivity are implicated in prosthetic hand control and can be optimized through gaze training. Journal of neuroengineering and rehabilitation. 2019;16(1):52.

7.	Parr JVV, Wright DJ, Uiga L, Marshall B, Mohamed MO, Wood G. A scoping review of the application of motor learning principles to optimize myoelectric prosthetic hand control. Prosthetics and orthotics international. 2021.

8.	Johnson L, Burridge JH, Demain SH. Internal and external focus of attention during gait re-education: an observational study of physical therapist practice in stroke rehabilitation. Physical therapy. 2013;93(7):957-66.

9.	Kim GJ, Hinojosa J, Rao AK, Batavia M, O'Dell MW. Randomized Trial on the Effects of Attentional Focus on Motor Training of the Upper Extremity Using Robotics With Individuals After Chronic Stroke. Archives of physical medicine and rehabilitation. 2017;98(10):1924-31.

10.	Kal E, van den Brink H, Houdijk H, van der Kamp J, Goossens PH, van Bennekom C, et al. How physical therapists instruct patients with stroke: an observational study on attentional focus during gait rehabilitation after stroke. Disabil Rehabil. 2017:1-12.

11.	Landers MR, Hatlevig RM, Davis AD, Richards AR, Rosenlof LE. Does attentional focus during balance training in people with Parkinson's disease affect outcome? A randomised controlled clinical trial. Clinical rehabilitation. 2016;30(1):53-63.

12.	Durham KF, Sackley CM, Wright CC, Wing AM, Edwards MG, van Vliet P. Attentional focus of feedback for improving performance of reach-to-grasp after stroke: a randomised crossover study. Physiotherapy. 2014;100(2):108-15.

13.	Chua LK, Wulf G, Lewthwaite R. Choose your words wisely: Optimizing impacts on standardized performance testing. Gait Posture. 2020;79:210-6.

14.	van der Graaff E, Hoozemans M, Pasteuning M, Veeger D, Beek PJ. Focus of attention instructions during baseball pitching training. Int J Sports Sci Coa. 2018;13(3):391-7.

15.	Diekfuss JA, Raisbeck LD. Focus of attention in NCAA division 1 collegiate athletes: A qualitative investigation. J Sport Exercise Psy. 2016;38:S58-S.

16.	Porter JM, Wu WWFW, Partridge JA. Focus of attention: Perspectives from elite track and field athletes at the 2007 USA Track and Field National Championships. J Sport Exercise Psy. 2009;31:S92-S3.

17.	Yamada M, Diekfuss JA, Raisbeck LD. Motor Behavior Literature Fails to Translate: A Preliminary Investigation into Coaching and Focus of Attention in Recreational Distance Runners. Int J Exerc Sci. 2020;13(5):789-801.

18.	Chua LK, Jimenez-Diaz J, Lewthwaite R, Kim T, Wulf G. Superiority of External Attentional Focus for Motor Performance and Learning: Systematic Reviews and Meta-Analyses. Psychological Bulletin. 2021;147(6):618-45.

19.	Yamada M, Higgins LQ, Raisbeck L. How external and internal focus are used in the field: A review. Int J Sports Sci Coa. 2021.
