# Supplementary material for: Direction of attentional focus in prosthetic training: Current practice and potential for improving motor learning in individuals with lower limb loss
Source: PLoS One. 2022 Jul 7;17(7):e0262977. doi: 10.1371/journal.pone.0262977 (PMC9262185; doi:10.1371/journal.pone.0262977)
Supplement: Attachment — Submitted filename: Response to reviewer comments v2.docx [file pone.0262977.s006.docx]

Response to authors

I thank the authors for making a great attempt to address my concerns. The structure and flow of the paper is much improved. However, there is still a more detail I would like in places, particularly regarding the analyses. I feel some of the responses have somewhat passively addressed my concerns and I worry that there is a misinterpretation of non-significant results. I would therefore appreciate further elaboration upon the following points:

**Thank you again for the thoughtful comments and suggestions. Your valuable input allowed us to clarify our key findings, that is prosthetists used predominantly internally-focused statements to provide instruction and feedback to their patients with amputation. Thanks to your careful review, we realized our original analysis and results descriptions were poorly organized and confusing. We have revised these sections accordingly.**

**We have responded to each one of your comments below. Our responses in this document is in bold and the revisions to the manuscript are highlighted in yellow.**

Authors:

*“As the focus of this study was observational and on lower limb prosthesis, we decided to put the Kristoffersen et al. studies in the discussion section to avoid overwhelming the readers, particularly clinicians, in the introduction. The following two references were added to line 270-273 in the discussion to highlight the need to understand the science underlying prosthetic training in response to the more sophisticated control requirements of modern prostheses.”*

RESPONSE:

My focus here was not primarily to highlight the Kristoffersen paper here per se. Rather, I was hoping the authors could provide some evidence in general to support the prediction that internal focus instructions would be highly frequent during rehabilitation. The authors make a good attempt to explain why internal focus instructions might be less effective, but little to suggest this practice would be common. I again feel as though the rationale for the study and the justification for your hypotheses could be strengthened by literature on the instructional content of rehabilitation practice. For example, is there any subjective data from therapists or patients across any domains that suggests a bias towards an internal focus? Indeed, you actually provide this information in the discussion. Could it be brought forward?

**Thank you for the clarification. We added the following statement to line 33-35:**

“In fact, it may be more common for prosthetists to use internally-focused instruction and feedback during gait training, as has been demonstrated in physical therapists treating patients with stroke and other practice fields.(1-3)”

**In the three references provided here, three studies demonstrated that internal focus instructions and/or feedback was more commonly used by physical therapists when treating patients with stroke, including during gait training. The Yamada et al. (2021) study is a recent comprehensive review on how attentional focus are used in various fields, in which the authors also concluded that internal focus is predominantly used by therapists and sports coaches.**

1. **Kal E, van den Brink H, Houdijk H, van der Kamp J, Goossens PH, van Bennekom C, et al. How physical therapists instruct patients with stroke: an observational study on attentional focus during gait rehabilitation after stroke. Disabil Rehabil. 2018;40(10):1154-65.**
2. **Johnson L, Burridge JH, Demain SH. Internal and external focus of attention during gait re-education: an observational study of physical therapist practice in stroke rehabilitation. Physical therapy. 2013;93(7):957-66.**
3. **Yamada M, Higgins LQ, Raisbeck L. How external and internal focus are used in the field: A review. Int J Sports Sci Coa. 2021.**
4. **Durham K, Van Vliet PM, Badger F, Sackley C. Use of information feedback and attentional focus of feedback in treating the person with a hemiplegic arm. Physiotherapy research international : the journal for researchers and clinicians in physical therapy. 2009;14(2):77-90.**

Authors:

*“Our primary justification for the study was that instructions to prosthetic device users may impact how well they learn to use the device. Clinical application of this theory has been demonstrated in several rehabilitation studies, which were cited here. To more clearly delineate our intention, we revised this sentence (line 46-49) to the following:”*

RESPONSE:

I believe my initial response was slightly too vague and therefore remains somewhat unanswered. To be specific, I want to know why the authors think IF instructions would be utilised less with higher function LLP users? Is there a specific rationale for this hypothesis? Currently it doesn’t seem justified. Are you suggesting that high functioning LLP users are more skilled ***because*** they are not receiving If instructions? Or are you suggesting that IF instructions are used more at the early stage of learning and less so when an LLP users becomes more skills? Either of these explanations need justification. Again, these points are better addressed in the discussion and therefore reads as though the hypotheses were derived post-hoc.

**Thank you for clarifying your comment. After much contemplating, we decided to remove the second hypothesis that stated internally focus would be utilized less when treating higher functioning LLP users. As this is the first study examining attentional focus in prosthesis users, there is no previous evidence to justify this hypothesis, and we believe removing this hypothesis would reduce the risk of confusing our readers.**

**The nature of our secondary purpose is exploratory. We intended to explore what factors may influence the use of attentional focus language during prosthetic training. An unjustified hypothesis here was unnecessary. Thank you for pointing that out.**

Authors:

*We provided the definitions and examples for “mixed focus” and “unfocused” statements in Table 1. Specifically, mixed focus was defined as: “A statement that includes both internal and external focus”, and unfocused statement was defined as: “A statement not giving technical instruction or offering encouragement to the learner only.”*

RESPONSE:

I was aware of the definitions in Table 1 but found it odd that only one type of instruction was defined in text. Is there a reason for this? Upon first reading I felt as though I would have benefited from all being defined in the same section.

**Thank you for the suggestion. The following sentence has been added:**

“Statements that included more than one type of focus (mixed focus) and without a specific focus (unfocused) were categorized separately (Table 1).”

Authors:

*Thank you for the suggestion. The Kruskal-Wallis tests were done to compare among the 6*

*prosthetists to see if there were any significant differences in their uses of internal and external*

*languages. The percentage for each type of statement used was aggregated for each prosthetist.*

RESPONSE:

Unfortunately, I am more confused now! If each type of statement were aggregated for each prosthetist, then you must have three values for each of the six prosthetists, one for IF, EF and mixed? You are then running the ANOVA to determine if there is a main effect of instruction type, and whether the group of six prosthetist tend to use one type of instruction more than another. If this is the case, then your design is technically repeated measures, and the Kruskal-Wallis is not suitable?

Alternatively, I don’t understand how you can run an analyses that compares each prosthetists individually to determine whether one prosthetist might use a type of instruction more than another?

I would need much more clarity here.

**Sorry about the confusion. Our purpose of running the Kruskal-Wallis test (non-parametric one-way ANOVA) was to examine whether there were differences in the utilization of the instruction types among the participating prosthetists. In other word, the prosthetists (6 levels) was the independent variable and the three % values (internal, external, and mixed) were the dependent variables. Three separate tests, one for each % value, were conducted.**

**This was an important first set of analyses for us, because we would like to ensure that the attentional focus usage pattern can be generalized among the clinicians in this study who had different training and experiential background. Our finding that there were no significant differences among the 6 clinicians (p=0.330-0.945, line 184-185) suggested that the usage pattern is similar among them.**

**To elaborate the reason behind this set of analyses, we added the following:**

“Due to the violation of normality assumption, the nonparametric Kruskal-Wallis tests were used to determine whether there were significant differences within the six participating prosthetists regarding the percentages of attentional focus statement types delivered. This analysis was conducted to examine if the attentional focus usage pattern can be generalized among the participating clinicians with different clinical training and experiential background.”

Author:

*Thank you for the suggestion. The Kruskal-Wallis tests were done to compare among the 6*

*prosthetists to see if there were any significant differences in their uses of internal and external*

*languages. The percentage for each type of statement used was aggregated for each prosthetist.*

*We revised this statement to the following to clarify:*

*“The nonparametric Kruskal-Wallis test was used to determine whether there was a significant difference in the attentional focus statement types delivered by the six participating prosthetists in the study.”*

*The revised sentence above now exactly corresponds with the following sentence in the results*

*section:*

*“There were no statistical differences in the types of attentional focus statements delivered by*

*the six prosthetists during the training sessions (p=0.330-0.945).”*

RESPONSE:

So to confirm, there was no significant different in the type of instruction delivered? This would suggest that IF instructions were not used any more frequently than EF and mixed instructions? Although the descriptive statistics suggest a bias towards IF instructions you inferential statistics do not? Unless I am mistaken this would prevent you from drawing the conclusion that

“Our hypothesis was confirmed that most of the verbal interactions delivered by prosthetists to LLP users were focused internally on the movements of the patients’ body and/or prosthesis, rather than externally on the intended movement effects.”

and

“Our results showed **a significant** but perhaps unsurprising trend that most (i.e. 63%)…”

Please can the authors explain this discrepancy?

**This is the key finding of our study, and we are glad you again pointed out the confusion in our descriptions of the statistical analysis and results. We can assure you that there indeed were significant differences in the types of instruction delivered by the participating prosthetists to their patients. Namely, the clinicians predominantly used internally focused statements (64%) over external (9%), mixed (3%), and unfocused (25%) during prosthetic training.**

**We believe the confusion was caused by the poor organization of our analyses and findings, specifically the purpose of the Kruskal-Wallis test was not explained well. Therefore, we revised the statistical analysis and results to the following to clarify these key points:**

**“**To compare the percentages of the four types of attentional focus statements delivered by the participating prosthetists to the LLP users, the mean percentage value and 95% confidence interval (95% CI) were computed for each focus type. Normality and homogeneity of variance assumptions were assessed using the Shapiro-Wilks test and White test, respectively. Due to the violation of normality assumption, the nonparametric Kruskal-Wallis tests were used to determine whether there were significant differences within the six participating prosthetists regarding the percentages of attentional focus statement types delivered. This analysis was conducted to examine if the attentional focus usage pattern can be generalized among the participating clinicians with different clinical training and experiential background.”

**“**Of all statements collected, 48% (436/904) were classified as internal focus of attention directed at the prosthesis or prosthetic component, and 15% (138/904) were classified as internal focus of attention directed at the LLP users’ body, residual limb, and intact limb. Overall, internally focused statements accounted for 64% of all verbal instructions and feedback (95% CI=60-67%, range=44-88%). Furthermore, 9% (77/904, 95% CI=7-10%) of the statements were classified as external focus of attention, 3% (30/904, 95% CI=2-4%) were classified as mixed focus of attention, and 25% (223/904, 95% CI=22-27%) were classified as unfocused. Given that the 95% CI of the internal focus proportion was significantly higher and did not overlap with that of any other focus types, we concluded that the participating prosthetists predominantly used instruction/feedback that invoked an internal focus of attention, particularly for directing LLP users’ attention to their prosthetic devices. There were no statistical differences within the six prosthetists regarding the types of attentional focus statements used (p=0.330-0.945).”

*Authors:*

*This secondary analysis was done to examine whether certain characteristics of the participants*

*(clinicians and patients) were predictive of greater use of the presumably less effective internal*

*focus instruction and feedback. We move this discussion to after the primary finding discussion,*

*and added the following sentence:*

RESPONSE:

I still feel the authors could attempt to speculate their finding regarding their finding that age and gender were related to frequency of IF. This would help better set up future work.

**Thank you for the suggestion. We expanded this discussion to the following two paragraphs:**

“In speculation that LLP users with certain characteristics may be biased to receive a greater proportion of the presumably less effective internally focused instruction and feedback, our secondary analysis examined these associations. We found that LLP users’ sex, age, and perceived mobility were significantly predictive of the percentage of internally-focused instructions and feedback they received. Specifically, women and older LLP users were more likely to receive a higher percentage of internal focus instructions. To the best of our knowledge, no other observational studies to date have reported similar sex and age biases regarding the use of attentional focus in clinical practice. Nevertheless, these biases may potentially impact the training and outcomes of patients, and should be examined further in the future.”

“Our findings also showed that LLP users with higher PLUS-M T-scores tend to receive a greater proportion of internal focus instructions. It is possible that when treating higher functioning LLP users, prosthetists may choose to focus on addressing subtle body or prosthetic component movements such as symmetry and other specific motor patterns. For example, many prosthetic knee components were designed with the expectation that the user would adopt a pronounced heel-to-toe weight-shifting pattern during the stance phase of gait(4-6), particularly during faster walking to allow rapid knee flexion and greater ground clearance.(7, 8) Internally-focused instructions and feedback may be easier and more intuitive to use when teaching these intricate skills to LLP users, leading to its more prevailing use in higher functioning patients. In a previous study by Kal et al. investigating the association between therapists’ attentional focus use in patients with stroke, they found that patients with a longer length of hospital stay tend to receive more external focus instruction and feedback. The authors suggested that the increase in the use of external focus may be a natural progression over the course of rehabilitation to fit the needs, goals, and functioning level of the patients.”

**References:**

1.	Yamada M, Higgins LQ, Raisbeck L. How external and internal focus are used in the field: A review. Int J Sports Sci Coa. 2021.

2.	Kal E, van den Brink H, Houdijk H, van der Kamp J, Goossens PH, van Bennekom C, et al. How physical therapists instruct patients with stroke: an observational study on attentional focus during gait rehabilitation after stroke. Disabil Rehabil. 2018;40(10):1154-65.

3.	Johnson L, Burridge JH, Demain SH. Internal and external focus of attention during gait re-education: an observational study of physical therapist practice in stroke rehabilitation. Physical therapy. 2013;93(7):957-66.

4.	Eberly VJ, Mulroy SJ, Gronley JK, Perry J, Yule WJ, Burnfield JM. Impact of a stance phase microprocessor-controlled knee prosthesis on level walking in lower functioning individuals with a transfemoral amputation. Prosthetics and orthotics international. 2014;38(6):447-55.

5.	Kaufman KR, Frittoli S, Frigo CA. Gait asymmetry of transfemoral amputees using mechanical and microprocessor-controlled prosthetic knees. Clinical biomechanics. 2012;27(5):460-5.

6.	Maaref K, Martinet N, Grumillier C, Ghannouchi S, Andre JM, Paysant J. Kinematics in the terminal swing phase of unilateral transfemoral amputees: microprocessor-controlled versus swing-phase control prosthetic knees. Archives of physical medicine and rehabilitation. 2010;91(6):919-25.

7.	Mohamed A, Sexton A, Simonsen K, McGibbon CA. Development of a Mechanistic Hypothesis Linking Compensatory Biomechanics and Stepping Asymmetry during Gait of Transfemoral Amputees. Appl Bionics Biomech. 2019;2019:4769242.

8.	Shojaei I, Hendershot BD, Wolf EJ, Bazrgari B. Persons with unilateral transfemoral amputation experience larger spinal loads during level-ground walking compared to able-bodied individuals. Clinical biomechanics. 2016;32:157-63.
